# Antimicrobial stewardship practices in Guatemala: communication, perceptions, and behaviors regarding antimicrobial prescribing

**DOI:** 10.1017/ash.2025.10089

**Published:** 2025-08-18

**Authors:** Dana R. Bowers, Clara Secaira, Nancy Sandoval, Mario Melgar, Nuria Chavez, Randall Lou-Meda, Herberth Maldonado, Brooke M. Ramay

**Affiliations:** 1 Department of Pharmacotherapy, Washington State University College of Pharmacy and Pharmaceutical Sciences, Yakima, WA, USA; 2 Center for Health Studies, Universidad del Valle de Guatemala, Guatemala City, Guatemala; 3 Hospital Roosevelt, Guatemala City, Guatemala; 4 Pediatric Infectious Diseases, Hospital Roosevelt, Guatemala City, Guatemala; 5 Hospital Regional de Zacapa, Zacapa, Guatemala; 6 Foundation for Children with Kidney Diseases – FUNDANIER-Hospital Roosevelt, Guatemala City, Guatemala; 7 Centro de Estudios en Salud, Universidad del Valle de Guatemala, Guatemala City, Guatemala; 8 Unidad de Cirugía Cardiovascular de Guatemala, Guatemala City, Guatemala; 9 Paul G Allen School of Global Health, Washington State University, Pullman, WA, USA

## Abstract

**Objective:**

To describe antimicrobial prescribing practices in 4 hospitals in Guatemala to guide the development of an ongoing antimicrobial stewardship (AS) project.

**Design:**

A cross-sectional mixed methodologies descriptive study design.

**Participants and setting:**

Practicing physicians from 4 hospitals (2 tertiary public hospitals and 2 specialty referral hospitals) within Guatemala City.

**Methods:**

All participants responded to a survey to ascertain 3 key areas of antimicrobial prescription practices: identify key players, communication among key players, and perceptions and behaviors regarding antimicrobial prescribing. A subset of respondents participated in semi-structured interviews to further explore experiences with AS team dynamics and communication.

**Results:**

One hundred and ten participants completed the survey (*n* = 110/145, 75.8%), and 79 completed the interview (*n* = 79/110, 71.8%). Antimicrobial prescribing is led by physicians who are responsible for maintaining communication with infectious disease physicians. The limited role of the pharmacist and the more predominant role of the microbiologist in antimicrobial selection were notable despite similar levels of training. Efficient communication about prescribing was perceived primarily among physicians, although existing hierarchies within the healthcare system negatively influenced decision-making strategies. Participants reported difficulty in choosing an antibiotic and indicated a preference for broad-spectrum antimicrobial use.

**Conclusions:**

The existing structure between physicians in hospitals facilitates antimicrobial prescribing practices. However, optimization of antimicrobial use may occur if multidisciplinary teams participate in antimicrobial selection activities. The results of this study provide valuable insight and can be used as a starting point toward the implementation of effective AS strategies within Guatemala and other similar countries in Central America and the Caribbean.

## Introduction

Antimicrobial resistance (AMR) is an ongoing problem. According to the World Health Organization, antimicrobial resistance is a leading cause of death worldwide.^1^ This is particularly problematic within resource-limited settings including developing countries. Recent literature estimates higher mortality rates attributed to antimicrobial resistant infections in developing countries, including Latin America and the Caribbean, compared to mortality from AMR related causes in higher income countries.^2,3^ Per a recent point prevalence survey of antibiotic use in 5 countries, antimicrobial use is also reportedly higher in Latin America where approximately 54% of hospitalized patients received at least 1 antibiotic, the 2 most commonly prescribed antibiotic classes included third-generation cephalosporins and carbapenems.^4^ Addressing widespread use of broad-spectrum antibiotics would likely have a significant impact on AMR in this setting, and although there is some literature about antimicrobial stewardship programs (ASP) in Latin American countries, the impact of these programs is not well documented.^5,6^

Documented high rates of antimicrobial use in developing countries, including Latin America, highlight the need for guidance from ASPs, yet these programs are not as common in Latin America in comparison to European countries and the United States.^4,5,7^ Barriers to effective ASP initiatives to guide prescribing practices have been previously explored by the Global Ministerial Summit on Patient Safety.^8^ Within developing countries, diagnostic challenges, knowledge or awareness of appropriate antimicrobial use, limited access to quality assured antibiotics, and inadequate healthcare facilities are barriers to effective ASP.^8^ Specific barriers for ASP in Latin America included limited leadership support, lack of dedicated staff, presence of power distance/hierarchical relationships, need for federal government support, and lack of implementation in smaller cities.^8^ Inability to effectively implement ASP programs can have detrimental effects, such as increased antimicrobial resistance through inappropriate antimicrobial use.^8^ Thus, it is important to identify country specific barriers toward implementation to effectively initiate ASP activities. Although there is some data about ASP implementation and impact within Latin America, data from Guatemala are sparse.^9,10^ We aimed to describe antimicrobial prescribing practices using a mixed methods approach in 4 hospitals in Guatemala. The purpose of the study was to generate evidence about communication structure, including barriers and facilitators, as well as perceptions and behaviors regarding antimicrobial prescribing to contribute to the development of an ongoing ASP.

## Methods

### Design

To address hospital antimicrobial prescription practices within Guatemala, a cross-sectional mixed methodologies descriptive study was carried out first, administering a knowledge, attitude, and practice (KAP) questionnaire, followed by semi-structured interviews.

### Setting

This study was carried out at 4 institutions in Guatemala participating in a quality improvement project that provided technical assistance for implementing ASPs, Smart Thrive on Antimicrobial Therapy in Guatemala (STAT-GT). Hospital Roosevelt and Hospital Regional Zacapa are public hospitals and have 1200 and 200 beds, respectively, and are both tertiary public hospitals. Unidad Nacional de Oncologia Pediatrica and Unidad de Cirugia Cardiovascular de Guatemala have 60 beds each and non-profit specialized referral hospitals in pediatric oncology and pediatric cardiac surgery, respectively. These hospitals were chosen by convenience, based on the availability of infectious diseases (ID) physicians to participate and their ability to work with hospital staff to develop an ASP. Furthermore, previous investigations of these hospitals revealed high use of broad-spectrum antibiotics indicating potential improvement of antimicrobial prescribing.^11^

### Participants

Physicians hired permanently or in medical residency positions from participating hospitals were invited and consented to participate in the study. Non-graduated medicine students or physicians in an internship/observership were excluded.

### Survey instrument

Using existing literature, a survey instrument was developed in REDCap (Research Electronic Data Capture) to ascertain baseline perceptions of antimicrobial stewardship practices.^12–15^ It included 46 questions regarding antimicrobial knowledge and resistance, prescribing practices, and multidisciplinary antimicrobial stewardship team acceptance (Appendix A). After informed consent was obtained, survey participants received an email with a brief introduction and the link to the survey. For non-responders, reminders were given via email and verbally by the researcher at each site. Survey data were collected from REDCAP, and descriptive statistics were performed on the quantitative data using SPSS software (Version 27, 2019).

### Semi-structured interviews

After completing the electronic survey, participants indicated their preferred time and day to conduct the telephone interview. Interviews were conducted in Spanish and included 6 open-ended questions to explore respondents’ experience with antimicrobial stewardship team dynamics and communication. Upon establishing contact with the participant, verbal consent and authorization to record the interview were provided. Seventy-nine of the 110 electronic survey participants completed the semi-structured interview.

Informed consent to record the interview was completed for each survey participant prior to beginning the interview. Audio files were transcribed into a double-entry matrix to code relevant themes. Patterns of similar responses and differences between responses were identified by the research team. Contextual quotes were translated to English and extracted from matrices to reflect the voices of the participants in the analysis. Then, verbatim quotes were triangulated with the quantitative results to reinforce the validity and reliability of the data collected.

The research was evaluated and approved by the Ethics Committee of the Center for Health Studies, Universidad del Valle de Guatemala (protocol number 220-11-2020) and by the Health Ethics Committee of the Ministry of Public Health and Social Assistance (protocol No. 31–020). At the local level, approval was received from the Research and Teaching Committees of the participating institutions.

## Results

### Participant demographics

Among the 4 institutions, 145 participants were enrolled, and 110 completed the electronic survey (75.8%, 110/145) (Table 1). Most survey participants were women (*n* = 59/110, 53.6%), and the average age of survey participants was 37 years (range 25–60 years) (Table 2). Thirty (*n* = 30/109, 27.5%) participants had a master’s degree, and 10 of 109 (9.2%) had a medical degree (Table 2). Most participants reported involvement in teaching activities at the undergraduate or graduate level (*n* = 61/110, 55.5%). The most commonly reported specialty area was internal medicine (*n* = 27/110, 24.5%) (Table 2).

**Table 1.** Number of respondents by participating hospital

	Beds	Enrolled (*N* = 145)	Completed questionnaires (*N* = 110)	Completed interviews (*N* = 79)
Hospital Roosevelt	1200	69	52	31
Unidad Nacional de Oncologia	60	20	16	15
Hospital Regional Zacapa	200	24	15	13
Unidad de Cirugia Cardiovascular	60	32	27	20

**Table 2.** Electronic survey participant demographics

	Survey item	*n* (%), *N* = 110
Age, sex	Female	59 (53.6%)
	Age, years mean (range)	37.2 (25–60)
Area within the hospital	Surgical ward	6 (5.5%)
	Internal medicine ward	27 (24.5%)
	Pediatrics ward	19 (17.3%)
	Emergency	5 (4.5%)
	Outpatient consultation	21 (19.1%)
	Orthopedics and trauma	4 (3.6%)
	Specialty consultant	4 (3.6%)
	Adult intensive care unit	8 (7.3%)
	Pediatric intensive care unit	9 (8.2%)
	Other	7 (6.4%)
	Involved in teaching activities	61 (55.5%)
Highest degree^*^	Medical degree (MD)	10 (9.2%)
	MD and master’s degree	30 (27.5%)
	MD and specialty	24 (22.0%)
	MD and subspecialty	24 (22.0%)
	MD in fellowship	21 (19.3%)

* *n* = 109.

### Semi-structured interviews

Of the 110 electronic survey participants, 79 completed the semi-structured interview. Most of the semi-structured interview participants were women (*n* = 40/79, 50.6%), and the average age was 37 years (range 25–60 years). In addition to being a certified medical doctor, some participants had a master´s degree (*n* = 20/79, 25.3%), and 7/79 (8.8%) had a bachelor´s degree in medicine.

#### Antimicrobial prescribing education

Of the 110 respondents to the electronic survey, physicians reported receiving antimicrobial prescribing education primarily during medical school and postgraduate specialty training (*n* = 89/110, 80.9% each). All 110 repondents reported to receive ongoing education, although they reported different educational modalities. When asked about ongoing education in antimicrobial prescribing within the previous year, 52.7% (*n* = 58/110) received education through their hospital department and 18.2% (*n* = 20/110) through independent coursework. For continuing education, most participants used the internet (50.9%, *n* = 56/110), information provided by higher-ranking physicians (50.0%, *n* = 55/110), and/or guidance from the Pan American Health Organization/World Health Organization (PAHO/WHO) for Antimicrobial Resistance (49.1%, *n* = 54/110).

#### Key players involved in antimicrobial prescribing

During semi-structured interviews, head physicians were identified as the main players in antimicrobial prescribing, whose main role was maintaining direct communication with the ID physician. An example of this relationship is demonstrated in the following quote:

“The infectious disease specialist is always consulted about the management of antibiotics, so in theory […], every prescribed antibiotic is reviewed by the infectious disease specialist.” (Male, 34 years old, public hospital)

Pharmacists were also identified for their role in antimicrobial guidance including medication dispensing and communicating any potential dosing or administration concerns to prescribing physicians, such as drug-drug interactions.

“First, the doctor must assess the antibiotic that is going to be prescribed based on the culture results or what antibiotics are on hand. One can also ask the pharmacist questions more related to the dosage […] because doctors can make mistakes when calculating doses, in which case the pharmacy can provide support.” (Woman, 35 years old, National Public Hospital)

#### Interprofessional communication

Most health professionals indicated that there is a lot of communication surrounding antimicrobial prescribing within their institutions (*n* = 100/106, 94.3%), describing communication as fluid and efficient. Further, prescribers were accessible to answer questions in person or by telephone, facilitating communication. Participants also reported strong interprofessional relationships among colleagues that continually facilitated communication (Table 3). These findings were also emphasized in open-ended interviews.

**Table 3.** Distribution of responses to selected electronic survey responses

Survey item	Response	Agreement, *n* (%), *N* = 110
The following professionals consistently communicate about antimicrobial prescribing	Between physicians and infectious disease physicians^*^	100 (94.3%)
	Between physicians^†^	99 (92.5%)
	Between physicians and pharmacists^†^	56 (52.3%)
	Between physicians and nurses^‡^	57 (54.8%)
Respondents are willing to receive feedback on antimicrobial prescribing from the following professionals	Infectious disease physicians^§^	107 (99.1%)
	Nurses^#^	33 (32.7%)
	Pharmacists^**^	69 (65.7%)
	Microbiologists^**^	96 (91.4%)
	A multidisciplinary team	106 (96.3%)
Antimicrobial prescribing recommendations are consistently accepted from the following professionals	A treating physician of senior rank	88 (80.0%)
	Other physicians^††^	64 (58.7%)
	Pharmacists	50 (45.5%)

* *N* = 106

† *N* = 107

‡ *N* = 104

§ *N* = 108

# *N* = 101

** *N* = 105

†† *N* = 109.

“I consider that [communication] is very good because, as I said, the infectious disease department is very attentive and frequently checks on patients from all the services, […] asking if there are new culture results or tests to review.” (Male, 31 years old, national public hospital)

Not all participants had a positive perception about communication regarding antimicrobials and expressed that, at times, it is ineffective. On some occasions, communication was interpreted as unidimensional and the result of established hierarchies between residents, attendings, and specialty physicians all of which created barriers to effective communication.

“It is not always the best communication, it should be an interaction that is multidirectional, but in many hospitals it is imposed and there are many barriers to communication, making it more difficult to prescribe. Sometimes higher-ranking physicians impose and say “this antibiotic is going to be given because I say so” and a consensus is not reached.” (Male, 31 years old, national public hospital)

#### Physician-to-physician communication

Participants elaborated on many factors that influence communication. The type of communication can vary depending on the department within the hospital or the hospital’s size. For example, respondents expressed that communication needs to be efficient and focused within the dynamic and fast-paced emergency department. Participants also mentioned the lack of clearly defined communication protocols, which hinders the effectiveness of communication. Lastly, the COVID-19 pandemic compounded existing communication issues through reliance on digital forms of communication. This made face-to-face communication difficult and influenced patient care.

The setting for communication was also discussed during the interviews. Most participants indicated that most communication occurs during patient rounds (*n* = 99/110, 90.0%) and is generally considered multidisciplinary and face-to-face. Another commonly reported communication form was exchanging information in medical chart notes (*n* = 94/110, 85.4%).

**Figure 1.**
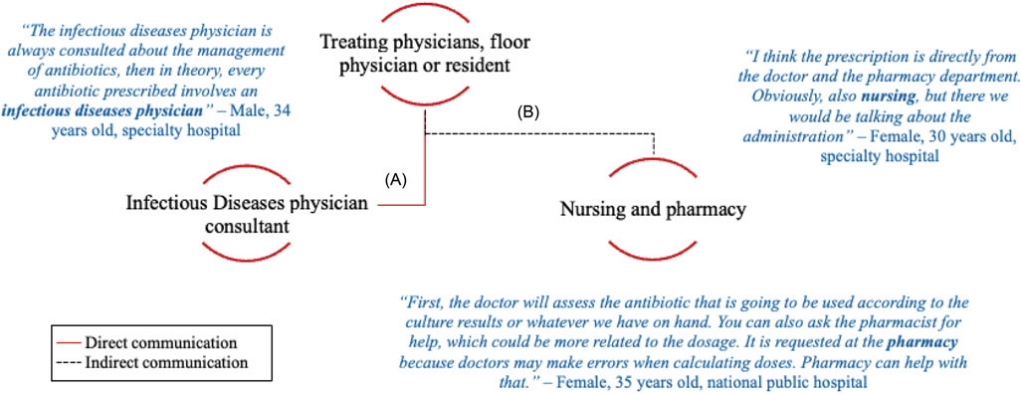
Types of direct and indirect communication physicians and non-physicians. Direct communication (A) occurs from physician to physician, while indirect communication (B) occurs between physicians and non-physicians such as nursing or pharmacy.

Physician-to-physician communication regarding antimicrobial prescribing was frequent and effective. Participants reported good communication with ID physicians, and 94.3% (*n* = 100/106) indicated that communication was effective. Participants also described effective communication between non-ID physicians (*n* = 99/107, 92.5%). Figure 1 shows direct communication between physicians, while indirect communication occurs with non-physicians such as nursing and pharmacy.

#### Non-physician-to-physician communication

Communication between pharmacists and physicians occurred infrequently (*n* = 56/107, 52.3%) and was similarly infrequent between nurses and physicians (*n* = 57/104, 54.8%) (Table 3).

#### Feedback

Participants were also asked about their willingness to accept feedback. Survey participants were most willing to accept feedback from ID physicians (*n* = 107/108, 99.1%) and multidisciplinary teams (*n* = 106/110, 96.3%). They were also willing to accept feedback from the microbiologists (*n* = 96/105, 91.4%). However, participants were less willing to receive feedback from pharmacists (*n* = 69/105, 65.7%) and nurses (*n* = 33/101, 32.7%) (Table 3).

“If it comes from a nurse or a pharmacist it is a little difficult, at least in our experience. Generally, the nurse does not handle much… at least here in Guatemala, nurses don’t handle therapeutic matters in general. With pharmacists, we have seen very few times that there has been contact or communication with them regarding this issue.” (Private hospital specialist, 25 years old, male)

### Perceptions and behaviors regarding antimicrobial prescribing

Antimicrobial prescribing perceptions and behaviors were explored within each institution. It identified that approximately 26.3% (*n* = 29/110) of participants’ decisions to prescribe antimicrobials are reviewed by another physician, and 17.2% (*n* = 19/110) discuss their decision to prescribe an antimicrobial with a senior colleague. When antimicrobial decisions are reviewed by a senior colleague, 90.0% (*n* = 99/110) of participants indicated that alternative antimicrobials are recommended instead of what was originally prescribed.

Most participants indicated that antimicrobial selection is difficult (45.5%, *n* = 50/110), though participants (*n* = 53/110, 48.1%) considered antimicrobial prescribing a shared decision. If there was uncertainty around antimicrobial selection, participants (*n* = 63/110, 57.2%) indicated their preference to prescribe broad-spectrum antimicrobials to ensure the possible infection was cured. If there was uncertainty about the bacterial versus non-bacterial pathology, 43.6% (*n* = 48/110) of participants indicated the preference to prescribe an antimicrobial rather than waiting for definitive results. Participants (25.4%, *n* = 28/110) indicated a preference for maximum dosages of antimicrobials when alternative antimicrobials were selected.

Monitoring for adverse events (AEs) of antimicrobials was also a concern for participants. When a patient experiences an AE, participants believe it indicates that there is perceived damage to the reputation of the treating physician (49.1% *n* = 54/110). If a patient clinically deteriorates, 81.8% (*n* = 90/110) of participants strongly agreed that there is a tendency to consider that the infection was not treated promptly. Finally, 88.2% (*n* = 97/110) considered that if an infection is not treated on time, it may result in a malpractice lawsuit.

Variability was also expressed in the optimal duration of antimicrobial therapy. A total of 24% (*n* = 26/110) of participants indicated continuing antimicrobials for a set duration was dependent on the type and severity of infection. Interestingly, 28.1% (*n* = 31/110) of participants indicated that the decision to discontinue an antimicrobial should always have the approval of their supervisor.

## Discussion

We aimed to describe antimicrobial prescribing practices using a mixed methods approach in 4 hospitals in Guatemala to generate evidence toward the development of an ongoing ASP.We show that physicians from hospitals included in this study make most decisions about antimicrobial prescribing by consulting among colleagues, or with ID physicians and less often by discussing antimicrobial interventions with microbiologists, pharmacists, and nurses. Limited collaboration with all healthcare professionals may be due to a lack of formal training in ASP development and antimicrobial prescribing among this group, the latter of which was disproportionately reported among the respondents. Participants reported receiving specialty training or continuing education during postgraduate education (27%) or from established agencies (17% from Pan American Health Organization). In Latin American countries, participation in training programs for the development of ASPs and IPCs is limited by lack of funds, protected time to receive education and insufficient staff to provide frequent training.^7,16^ Even in areas where resources to invest in education and development of stewardship programs are available, the “education” domain has been reported to be the most deficient during self-assessment exercises carried out in 77 hospitals in 9 Latin American countries.^7^ Targeted educational programs delivered by dedicated personnel may improve the effectiveness of ASPs and antimicrobial prescribing activities but should consider specific cultural and contextual challenges faced Latin America.^10,17^

Few respondents reported effective communication occurring between physicians and pharmacists (52.3%) or nurses (54.8%). In contrast, effective communication reported by respondents was much higher with ID physicians (94.3%) and non-ID physicians (92.5.%) and may be indicative of hierarchical relationships occurring in communication. Effective communication is essential to achieve optimal patient care but may be limited by barriers that may be cultural, hierarchical, and personality-dependent.^18^ This was emphasized in a recent study where hierarchical relationships hindered effective multidisciplinary efforts.^10^ Development of formalized, multidisciplinary approaches to ASP may promote trusting relationships capable of overcoming hierarchical relationships.

Providers indicated a strong tendency to receive feedback on the prescription of antimicrobials from microbiologists (91.4%) in comparison to a lower proportion of respondents communicating with pharmacists (65.7%) and nurses (32.7%) (Table 3). Prescribers are likely more inclined to discuss prescribing with microbiologists because they have access to clinical data. In contrast, pharmacists in Guatemala are primarily involved in dispensing activities. In addition, disciplinary power distance, as a result of limited clinical training, restricts the breadth of services pharmacists provide within this context.^19^ Many participants expressed antibiotic prescribing hesitancy in cases of clinical uncertainty, the latter of which has been shown to influence clinical decisions around prescribing.^20^ This uncertainty can lead to prescribing antibiotics to treat non-bacterial infections “just in case.” This is expected given few respondents were familiar with available protocols to assist with antimicrobial prescribing. It is important to note that few hospitals have published protocols to guide antimicrobial use, which may explain unfamiliarity. Nevertheless, many international guidelines are available, such as the World Health Organization, which provides a toolkit specifically for developing countries.^12^ Fabre et al. describe similar findings in Latin America, indicating that guideline use may be improved with more effective collaboration among healthcare workers to facilitate the exchange of ideas and share references, albeit international, that may support antimicrobial prescribing.^19^

Physicians indicated concerns about patient outcomes after antibiotic use, either as a result of adverse effects or improperly treated infections. One way to address these concerns may be through the development of standardized protocols using data from local hospital-level antibiograms to support the development of stewardship programs. At a national level, the development of action plans may improve the monitoring and assessment of strategies for antimicrobial resistance.^21^ Avello et al surveyed national action plans (NAP) for antimicrobial resistance within Latin America and showed that during the survey time frame of 2020–2021, only 15 Latin American countries had an approved NAP.^21^ Guatemala did not have an approved NAP at the time of this study but has since initiated development (2024) and requires ongoing support from the Ministry of Health to approve policies to address ongoing antimicrobial resistance and stewardship efforts.

Limitations of this study include simultaneous data collection from multiple, large medical institutions and assumptions that questions were interpreted uniformly by providers from multiple study sites. Additionally, the facilities included in this study may not be representative of all variations in practices within Guatemala. Nevertheless, the results of this study provide valuable insight into the knowledge, attitudes, and current prescribing behaviors regarding antimicrobials and ASP practices within Guatemala. Deficiencies in confidence around clinical knowledge, rigid prescribing perceptions, hierarchical relationships, and barriers to effective multidisciplinary communication were elucidated.

## Conclusion

The existing structure between physicians in hospitals facilitates antimicrobial prescribing practices. However, optimization of antimicrobial use may occur if multidisciplinary teams participate in antimicrobial selection activities. The results of this study can be used as a starting point to implement effective ASP strategies within Guatemala and potentially other Latin American countries.

## Supporting information

10.1017/ash.2025.10089.sm001 Bowers et al. supplementary material 1 Bowers et al. supplementary material

10.1017/ash.2025.10089.sm002 Bowers et al. supplementary material 2 Bowers et al. supplementary material

## Supplementary materials

The supplementary material for this article can be found at https://doi.org/10.1017/ash.2025.10089.
