# Supplementary material for: Antimicrobial stewardship practices in Guatemala: communication, perceptions, and behaviors regarding antimicrobial prescribing
Source: Antimicrob Steward Healthc Epidemiol. 2025 Aug 18;5(1):e188. doi: 10.1017/ash.2025.10089 (PMC12394028; doi:10.1017/ash.2025.10089)
Supplement: Bowers et al. supplementary material 1 — Bowers et al. supplementary material [file S2732494X25100892sup001.docx]

**Knowledge, Attitudes and Practices on Antimicrobial Prescribing by Physicians in the Hospital Setting**

**Please complete the following questionnaire and submit by clicking the Submit button at the end.**

**Only one response per participant is accepted. If you accidentally close your browser you will need to complete the questionnaire again. If you are unable to complete it now you can open the link at any time.**

**I. PARTICIPANT INFORMATION**

**1. What is your age in years?**

- **(Years)**

**2. What is your gender?**

- **Female**
- **Male**

**3. What is your level of education?**

- **Bachelor's Degree**
- **Master's Degree Specialty**
- **Medical subspecialty Fellow (in training)**

**4. Specialization/subspecialization/fellow (Check all that apply)**

**Allergy**

**Anesthesiology**

**Cardiology**

**Cardiac surgery**

**General surgery**

**Oral and maxillofacial surgery**

**Orthopedic surgery**

**Pediatric surgery**

**Plastic surgery**

**Thoracic surgery**

**Dermatology**

**Endocrinology**

**Epidemiology**

**Pharmacology**

**Gastroenterology**

**Medical genetics**

**Geriatrics**

**Gynecology and Obstetrics**

**Hematology**

**Infectious diseses**

**Emergency medicine**

**Sports medicine**

**Occupational medicine**

**Family and community medicine**

**Physical medicine and rehabilitation**

**Forensic medicine**

**General medicine**

**Intensive medicine**

**Internal medicine**

**Preventive medicine in public health**

**Microbiology**

**Nephrology**

**Pulmonology**

**Neurosurgery**

**Clinical neurophysiology**

**Neurology**

**Nutrition Ophthalmology**

**Medical oncology**

**Radiation oncology**

**Otorhinolaryngology**

**Pediatrics**

**Psychiatry**

**Radiology**

**Rheumatology**

**Toxicology**

**Traumatology**

**Urology**

**Family and community medicine**

**Physical medicine and rehabilitation Other**

**4.1 Other Specialization/subspecialization/fellowship (specify)**

**5. How many years have you practiced medicine since you obtained your medical and surgical degree?**

**6. Are you involved in teaching activities?**

- **Yes**
- **No**

**6.1 If involved, Degree Postgraduate**

**7. In which hospital do you currently work?**

- **Cardiovascular Surgery Unit of Guatemala (UNICAR)**
- **Roosevelt Hospital**
- **Pediatric Oncology Unit (UNOP) FUNDANIER**
- **Zacapa Regional Hospital Other**

**7.1 Other (specify)**

**8. In which area of ​​the hospital do you carry out most of your activities?**

- **Surgery**
- **Internal medicine**
- **Pediatric**
- **Emergency**
- **Gynecology and obstetrics**
- **Outpatient clinic**
- **Orthopedics**
- **Specialist consultant**
- **Adult intensive care unit**
- **Pediatric intensive care unit**
- **Other**

**8.1 Other area (specify)**

**9. How many hours does your workday at the hospital last**

**10. Approximately how many patients do you see in a typical workday?**

**11. Approximately how much time in minutes do you spend caring for a patient?**

**II. KNOWLEDGE ABOUT ANTIMICROBIALS**

**Training and education on the use of antimicrobials**

**1) At what point in your training did you receive training in prescribing antimicrobials? Check all that apply.**

- **Medical school**
- **Medical conferences**
- **Course or workshop**
- **Diploma**
- **During specialty training**
- **During subspecialty training**
- **Online courses**
- **Other**

**1.1. Other (please specify)**

**2. In 2019, how many times did you receive any type of education on antimicrobial use?**

**None**

- **At least once**
- **Between one and three times**
- **More than three times**

**3. Where were these teachings given? (Check all that apply)**

- **Academic activities of your department**
- **Participation in independent courses**
- **Medical education**
- **Visit passes**
- **Webinars**
- **Other**

**3.1 other (please specify)**

**4. Which of the following sources of information do you use as part of your continuing medical education on antimicrobial use? (Check all that apply)**

- **Information shared by higher-ranking physicians**
- **Information shared by other physicians (of the same rank)**
- **Internet**
- **Mobile App**
- **National guidelines for the diagnosis and treatment of diseases PAHO/WHO Antimicrobial Guide**
- **Other Treatment Guide Others**

**4.1 Name of websites (specify)**

**4.2 Name of mobile apps (specify)**

**4.3 Name of guidelines (specify)**

**4.4 Others (specify)**

**5. How widely available do you consider information on optimal use of antimicrobials to be?**

- **Very widely available**
- **Available**
- **Not available**

**III. PRACTICES ON PRESCRIBING ANTIMICROBIALS**

**Prescribing antimicrobials**

**6. To which population do you most frequently prescribe antimicrobials?**

- **Neonates**
- **Pediatrics**
- **Adults**
- **Elderly**

**7. How frequently do you prescribe antimicrobials in your clinical practice?**

- **Every day**
- **Most days**
- **Some days**
- **Rarely**

**8. What type of antimicrobials do you most often prescribe in your daily practice? (Matrix Question - Never, Once a month, One to three times a month, At least once a week)**

- **Aminoglycosides (e.g. gentamicin, amikacin)**
- **Broad spectrum beta-lactamsspectrum (piperacillin tazobactam)**
- **Narrow-spectrum beta-lactams (amoxicillin, ampicillin)**
- **Carbapenems (e.g. Ertapenem, meropenem)**
- **Broad-spectrum cephalosporins (e.g. Cefepime, Ceftriaxone, Cefotaxime)**
- **Narrow-spectrum cephalosporins (e.g. Cephalothin, Cefazolin)**
- **Clindamycin**
- **Fluoroquinolones (e.g. Ciprofloxacin, Levofloxacin)**
- **Vancomycin**
- **Antifungals (e.g. Fluconazole, Amphotericin)**

**9. In your opinion, should a third-line antimicrobial (such as a carbapenem) be prescribed until microbiological culture results are available?**

- **Strongly agree**
- **Agree**
- **Disagree**
- **Strongly disagree**

**Key Players involved in prescribing antimicrobials**

**10. Regarding the prescription of antimicrobials, how much communication do you think exists between the following professionals? (Matrix Question - A lot , A little, Very little, None**

- **Between doctors**
- **Between doctors and pharmacists Between doctors and nurses**
- **Between doctors and infectious disease specialist**

**11. How do different health professionals communicate about the prescription of antimicrobials?**

**a. Direct communication:**

- **In-person interaction**
- **Telephone**
- **Text messages**
- **Consultations with the infectious disease specialist**

**b. Passive forms:**

- **Notes in the records**
- **Warning/alert systems**
- **Other (open answer)**

**b1. Other passive forms (specify)**

**c. In groups:**

- **Visiting passes**
- **Informal meetings of colleagues (in the hallway)**
- **Other (open answer)**

**c1. Other group (specify)**

**12. During the process of prescribing an antimicrobial, is there communication with the patient to inform him/her about the use of antibiotics? (Or with the mother or father in case the patient is a minor)**

- **Yes**
- **No**

**12.1 Why do you think there is no communication with the patient when prescribing an antibiotic?**

**13. What type of information is communicated to the patient?**

- **Indication**
- **Adverse effects**
- **Duration of treatment**

**14. When discharging a patient, who is responsible for giving instructions to patients about the use of antimicrobials? (Administration times, what to expect from the medication, how you will feel, etc.)**

- **Nurse**
- **Doctor**
- **Pharmacist**
- **None**

**15. How often does someone within the hospital supervise your decision to prescribe an antimicrobial? (administration times, dosage, type of antimicrobial, etc.)**

- **Never**
- **Sometimes**
- **Most of the time**
- **Always**

**16. How often do you review your decision to prescribe an antimicrobial with a more senior colleague? (By your own decision)**

- **Never**
- **Sometimes**
- **Most of the time**
- **Always**

**17. If you review your decision with a more senior colleague, how often does your colleague recommend antimicrobials other than those you would have liked to prescribe?**

- **Never**
- **Sometimes**
- **Most of the time**
- **Always**

**Confidence regarding prescribing an antimicrobial**

**18. Do you think it is difficult to select the correct antimicrobial?**

- **Strongly agree**
- **Agree**
- **Disagree**
- **Strongly disagree**

**19. When prescribing an antimicrobial, how confident are you regarding the following specifications?**

- **Very confident**
- **Somewhat confident**
- **Slightly confident**
- **Not at all confident**

**a. Type of antimicrobial prescribed**

**b. Selection of antimicrobial based on microbiological results**

**c. Dose of antimicrobial prescribed**

**d. Duration of antimicrobial prescribed**

**e. Dosage and interval adjustment in cases of renal dysfunction**

**20. To which population do you most frequently prescribe antimicrobials?**

- **Neonates**
- **Pediatrics**
- **Adults**
- **Elderly**

**Please answer the following questions Yes/No**

**21. In cases of doubt, do you consider it preferable to use a broad-spectrum antimicrobial to ensure that the possible infection is cured?**

**22. In situations where it is difficult for you to closely monitor your patients, do you consider that you frequently prescribe antimicrobials?**

**23. In situations where there is doubt that it is a bacterial infection, do you consider it better to prescribe an antimicrobial?**

**24. If the antimicrobial to be used is not the original molecule, do you prefer to use the maximum dose?**

**25. Do you consider that an infection not treated in time can result in a malpractice lawsuit?**

**Please indicate how much you agree with the following statements (Strongly agree, Agree, Disagree Strongly, disagree)**

**26. If a patient's condition deteriorates, there is a tendency to consider that the reason is an infection that has not been promptly treated**

**27. If a patient suffers adverse events, the reputation of the doctor treating him/her is perceived as being damaged**

**28. It is not acceptable for the head physician to give his/her opinion or suggestions about the antimicrobials that are being prescribed**

**29. It is not acceptable for another physician to modify the antimicrobials**

**or stop a patient's antimicrobial treatment**

**30. It is not recommended that a pharmacist suggest a change in antimicrobial**

**31. In my institution, the withdrawal of antimicrobials is not accepted until a predetermined number of days have been completed**

**32. The decision to stop an antimicrobial almost always has to be approved by my boss**

**33. The selection of the antimicrobial to prescribe is a shared decision**

**Please select the correct answer (you can check more than one option)**

**34. Antibiotics are included in the treatment protocol for COVID-19. Why?**

**a. To eliminate the coronavirus.**

**b. To treat secondary infections**

**c. To prevent collateral infections**

**d. None of these is correct**

**IV ACCEPTANCE OF INTERDISCIPLINARY TEAM**

**35. Do you think that doctors are willing to receive feedback from other health professionals to optimize the prescription of antimicrobials?**

- **Strongly agree**
- **Agree**
- **Disagree**
- **Strongly disagree**

**36. From the following list of health professionals, establish the order of who the doctor would be most willing to receive feedback when prescribing an antimicrobial (from very willing to not at all willing).**

- **Nurse**
- **Pharmacist**
- **Microbiologist**
- **Infectologist**

**37. If a multidisciplinary infectious disease team, as part of a strategy, reviews antibiotic therapy and suggests a modification, do you think you would be willing to consider the modification? Strongly agree**

- **Agree**
- **Disagree**
- **Strongly disagree**

**V ANTIMICROBIAL RESISTANCE**

**38. Antimicrobials are overused in Guatemalan hospitals**

- **Strongly agree**
- **Agree**
- **Disagree**
- **Strongly disagree**

**39. Antimicrobials may stop working in the future**

- **Strongly agree**
- **Agree**
- **Disagree**
- **Strongly disagree**

**40. Do you know what antimicrobial resistance is?**

- **Yes**
- **No**

**41. Where and when did you learn about antimicrobial resistance?**

**42. If you answered that you know what antimicrobial resistance is, could you tell me in your own words what antimicrobial resistance is?**

**Answer yes or no to the following questions:**

**43. Do you think that since you learned about antimicrobial resistance your antibiotic prescribing habits have changed?**

**44. Do you think that antimicrobial resistance is a worldwide problem?**

**45. Do you think that antimicrobial resistance is a national problem?**

**46. Do you consider that antimicrobial resistance is a problem in your daily practice?**

**47. Do you consider that your prescription contributes to generating antimicrobial resistance?**

**You have reached the end of the questionnaire, we thank you for your participation.**

**Below please indicate two options for the day and time when we can contact you. Someone from the research team will contact you to ask you the open questions in the questionnaire. The call will not take more than 20 minutes.**
