# Supplementary material for: Antimicrobial stewardship practices in Guatemala: communication, perceptions, and behaviors regarding antimicrobial prescribing
Source: Antimicrob Steward Healthc Epidemiol. 2025 Aug 18;5(1):e188. doi: 10.1017/ash.2025.10089 (PMC12394028; doi:10.1017/ash.2025.10089)
Supplement: Bowers et al. supplementary material 2 — Bowers et al. supplementary material [file S2732494X25100892sup002.pdf]

Conocimientos, Actitudes y Prácticas sobre la prescripción de antimicrobianos por médicos en el contexto hospitalario

Por favor responda al siguiente cuestionario y haga su envío pulsando el botón Submit (enviar) al final.

Solo se acepta una respuesta por participante.Si por error cierra su navegador será necesario volver a completar el cuestionario nuevamente. Si no puede completarlo ahora puede abrir el enlace en cualquier momento.

I. INFORMACIÓN DE EL O LA PARTICIPANTE

1. ¿Cuál es su edad en años?

____
(Años)

2. ¿Cuál es su género?

◯ Femenino ◯ Masculino

3. ¿Cuál es su nivel de formación?

◯ Licenciatura
◯ Maestría
◯ Especialidad
◯ Subespecialidad médica
◯ Fellow (en entrenamiento)

4. Especialización/subespecialización/fellow (Marque las que apliquen)

☐ Alergología
☐ Anatomía patológica
☐ Anestesiología
☐ Cardiología
☐ Cirugía cardíaca
☐ Cirugía general
☐ Cirugía oral y maxilofacial
☐ Cirugía ortopédica
☐ Cirugía pediátrica
☐ Cirugía plástica
☐ Cirugía torácica
☐ Dermatología
☐ Endocrinología
☐ Epidemiología
☐ Farmacología
☐ Gastroenterología
☐ Genética médica
☐ Geriatría
☐ Ginecología y Obstetricia
☐ Hematología
☐ Infectología
☐ Medicina de Urgencias
☐ Medicina del deporte
☐ Medicina del trabajo
☐ Medicina familiar y comunitaria
☐ Medicina física y rehabilitación
☐ Medicina forense
☐ Medicina general
☐ Medicina intensiva
☐ Medicina interna
☐ Medicina preventiva en salud pública
☐ Microbiología
☐ Nefrología
☐ Neumología
☐ Neurocirugía
☐ Neurofisiología clínica
☐ Neurología
☐ Nutriología
☐ Oftalmología
☐ Oncología médica
☐ Oncología radioterapéutica
☐ Otorrinolaringología
☐ Pediatría
☐ Psiquiatría
☐ Radiología
☐ Reumatología
☐ Toxicología
☐ Traumatología
☐ Urología
☐ Medicina familiar y comunitaria
☐ Medicina física y rehabilitación
☐ Otra

4.1 Otra Especialización/subespecialización/fellow (especifique)

____

5. ¿Cuántos años ha practicado medicina desde que obtuvo su título de médico y cirujano?

____

6. ¿Está involucrado en actividades de docencia/enseñanza?

◯ Sí ◯ No

6.1 Si está involucrado,

☐ Grado ☐ Postgrado

7. ¿En qué hospital labora actualmente?

☐ Unidad de Cirugía Cardiovascular de Guatemala (UNICAR)
☐ Hospital Roosevelt
☐ Unidad de Oncología Pediátrica (UNOP)
☐ FUNDANIER
☐ Hospital Regional de Zacapa
☐ Otro

7.1 Otro (especifique)

____

8. ¿En qué área del hospital desempeña la mayor parte de sus actividades?

◯ Cirugía
◯ Encamamiento medicina interna
◯ Encamamiento de pediatría
◯ Emergencia
◯ Ginecología y obstetricia
◯ Consulta externa
◯ Ortopedia y traumatología
◯ Interconsultante de especialidad
◯ Unidad de cuidados intensivos de adultos
◯ Unidad de cuidados intensivos de pediatría
◯ Otra

8.1 Otra área (especifique)

____

9. ¿Cuántas horas tiene su jornada de trabajo en el hospital?

____

10. ¿Aproximadamente cuántos pacientes ve en una jornada de trabajo ordinaria?

____

11. ¿Aproximadamente cuánto tiempo en minutos dedica a la atención de un paciente?

____

II. CONOCIMIENTOS SOBRE ANTIMICROBIANOS

Formación y educación sobre el uso de antimicrobianos

1) ¿En qué momento de su formación ha recibido entrenamiento para la prescripción de antimicrobianos? Marque todas las que apliquen.

☐ Facultad de medicina
☐ Congresos médicos
☐ Curso o taller
☐ Diplomado
☐ Durante el entrenamiento de especialidad
☐ Durante el entrenamiento de subespecialidad
☐ Cursos en línea
☐ Otro

1.1. Otro (especifique)

____

2. En el año 2019, ¿Cuántas veces recibió algún tipo de enseñanza sobre uso de antimicrobianos?

◯ Ninguna
◯ Al menos una vez
◯ Entre una y tres veces
◯ Más de tres veces

3. ¿En dónde se impartieron estas enseñanzas? (Marque todas las que apliquen)

☐ Actividades académicas de su departamento
☐ Participación en cursos independientes
☐ Educación médica
☐ Pases de visita
☐ Webinars
☐ Otro

3.1 otro (especifique)

____

4. ¿Cuál de las siguientes fuentes de información utiliza como parte de su educación médica continua sobre el uso de antimicrobianos? (Marque todas las que apliquen)

☐ Información compartida por médicos de mayor rango
☐ Información compartida por otros médicos (del mismo rango)
☐ Internet
☐ Aplicación Móvil
☐ Guías nacionales para el diagnóstico y tratamiento de enfermedades
☐ Guía de antimicrobianos de la OPS/OMS
☐ Otra Guía de tratamiento
☐ Otros

4.1 Nombre de los sitios Web (especifique)

4.2 Nombre de las aplicaciones móviles (especifique)

4.3 Nombre de las guías (especifique)

4.4 Otros (especifique)

5. ¿Qué tan disponible considera que se encuentra la información sobre el uso óptimo de antimicrobianos?

◯ Muy disponible
◯ Disponible
◯ No disponible
◯ Nada disponible

III. PRÁCTICAS SOBRE LA PRESCRIPCIÓN DE ANTIMICROBIANOS

Prescripción de antimicrobianos

6. ¿A qué población le prescribe antimicrobianos con mayor frecuencia?

◯ Neonatos
◯ Pediatría
◯ Adulto
◯ Adulto mayor

7. ¿Qué tan frecuentemente prescribe antimicrobianos en su práctica clínica?

◯ Todos los días
◯ La mayor parte de días
◯ Algunos días
◯ Raras veces

8. ¿Qué tipo de antimicrobianos son los que más prescribe en su práctica diaria?

	Nunca	Una vez al mes	Entre una y tres veces al mes	Al menos una vez por semana
Aminoglucósidos (p.ej. Gentamicina, amikacina)	◯	◯	◯	◯
Betalactámicos de amplio espectro (piperacilina tazobactam)	◯	◯	◯	◯
Betalactámicos de bajo espectro (amoxicilina, ampicilina)	◯	◯	◯	◯
Carbapenémicos (p. ej. Ertapenem, meropenem)	◯	◯	◯	◯
Cefalosporinas de amplio espectro (p.ej. Cefepime, Ceftriaxona, cefotaxima)	◯	◯	◯	◯
Cefalosporinas de bajo espectro (p.ej. Cefalotina, cefazolina)	◯	◯	◯	◯

Clindamicina	◯	◯	◯	◯
Fluoroquinolonas (p. ej. Ciprofloxacina, levofloxacina)	◯	◯	◯	◯
Vancomicina	◯	◯	◯	◯
Antifúngicos (p. ej. Fluconazol, anfotericina)	◯	◯	◯	◯

9. En su opinión, ¿un antimicrobiano de tercera línea (como un carbapenémico) debería ser prescrito hasta tener resultados de un cultivo microbiológico?

◯ Muy de acuerdo
◯ De acuerdo
◯ En desacuerdo
◯ Muy en desacuerdo

Actores involucrados en la prescripción de antimicrobianos

---

10. En relación a la prescripción de antimicrobiano ¿Cuánta comunicación considera que existe entre los siguientes profesionales?

	Mucha	Poca	Muy poca	Nula
Entre médicos	◯	◯	◯	◯
Entre médicos y farmacéuticos	◯	◯	◯	◯
Entre médicos y enfermeras	◯	◯	◯	◯
Entre médicos y médico infectólogo	◯	◯	◯	◯

11. ¿De qué forma se comunican los diferentes profesionales en salud sobre la prescripción de antimicrobianos?

a. Comunicación directa:

☐ Interacción en persona
☐ Teléfono
☐ Mensajes de texto
☐ Consultas con el infectólogo

b. Formas pasivas:

☐ Notas en los expedientes
☐ Sistemas de aviso/alerta
☐ Otras (respuesta abierta)

b1. Otras formas pasivas (especifique)

c. En grupos:

☐ Pases de visita
☐ Reuniones de colegas informales (en el pasillo)
☐ Otro (respuesta abierta)

c1. Otro grupo (especifique)

12. Durante el proceso de recetar un antimicrobiano, ¿Existe comunicación con el/la paciente para informarle sobre el uso de antibiótico? (O con la madre o el padre en caso de que el/la paciente sea menor de edad)

◯ Sí ◯ No

12.1 ¿Por qué cree que no existe comunicación con el/la paciente al momento de prescribirle un antibiótico?

13. ¿Qué tipo de información se le comunica al paciente?

☐ Indicación
☐ Efectos adversos
☐ Duración del tratamiento

14. Al darle de alta a un paciente ¿Quién se encarga de darle las indicaciones a los/las pacientes sobre el uso de antimicrobianos? (Tiempos de administración, qué esperar del medicamento, cómo se sentirá, etc.)

◯ Enfermera
◯ Médico
◯ Farmacéutico
◯ Ninguno

15. ¿Con qué frecuencia alguien dentro del hospital supervisa su decisión de recetar un antimicrobiano? (tiempos de administración, dosis, tipo de antimicrobiano, etc.).

◯ Nunca
◯ Algunas veces
◯ La mayoría de las veces
◯ Siempre

16. ¿Con qué frecuencia revisa su decisión de recetar un antimicrobiano con un colega de mayor rango? (Por decisión propia)

◯ Nunca
◯ Algunas veces
◯ La mayoría de las veces
◯ Siempre

17. Si usted revisa su decisión con un colega mayor en rango, ¿Con qué frecuencia su colega le recomienda antimicrobianos diferentes a aquellos que usted le hubiese gustado prescribir?

◯ Nunca
◯ Algunas veces
◯ La mayoría de las veces
◯ Siempre

Confianza respecto a la prescripción de un antimicrobiano

18. Considera que es difícil seleccionar el antimicrobiano correcto?

◯ Muy de acuerdo
◯ De acuerdo
◯ En desacuerdo
◯ Muy en desacuerdo

19. Cuando receta un antimicrobiano, ¿Qué tanta confianza tiene respecto a las siguientes especificaciones?

	Mucha confianza	Algo de confianza	Poca confianza	Nada de confianza
a. Tipo de antimicrobiano que receta	◯	◯	◯	◯
b. Selección de antimicrobiano con base a los resultados microbiológicos	◯	◯	◯	◯
c. Dosis de antimicrobiano que receta	◯	◯	◯	◯
d. Duración del antimicrobiano	◯	◯	◯	◯
e. Ajuste de dosis e intervalo en los casos de disfunción renal	◯	◯	◯	◯

20. ¿A qué población le prescribe antimicrobianos con mayor frecuencia?

◯ Neonatos
◯ Pediatría
◯ Adulto
◯ Adulto mayor

Por favor responda las siguientes preguntas de Sí/No

21. En los casos de duda, ¿Considera que es preferible utilizar un antimicrobiano de amplio espectro para asegurar que la posible infección sea curada?

◯ Sí
◯ No

22. ¿En las situaciones en donde se le es difícil dar un seguimiento cercano a sus pacientes, ¿Considera que receta frecuentemente antimicrobianos?

◯ Sí
◯ No

23. En las situaciones en donde existe duda de que se trate de una infección bacteriana, ¿Considera que es mejor prescribir un antimicrobiano?

◯ Sí
◯ No

24. Si el antimicrobiano a utilizar no es la molécula original, ¿Prefiere utilizar la dosis máxima?

◯ Sí
◯ No

25.¿Considera que una infección no tratada a tiempo puede resultar en una demanda por mala práctica?

◯ Sí
◯ No

Por favor indique qué tan de acuerdo se encuentra con los siguientes enunciados.

26. Si un paciente deteriora su condición, existe una tendencia de considerar que la razón es una infección que no ha sido oportunamente tratada

◯ Muy de acuerdo
◯ De acuerdo
◯ En desacuerdo
◯ Muy en desacuerdo

27. Si un paciente sufre eventos adversos, se percibe un daño a la reputación del médico que lo está tratando

◯ Muy de acuerdo
◯ De acuerdo
◯ En desacuerdo
◯ Muy en desacuerdo

28. No es aceptable que opine o sugiera a el médico jefe sobre los antimicrobianos que se están prescribiendo

◯ Muy de acuerdo
◯ De acuerdo
◯ En desacuerdo
◯ Muy en desacuerdo

29. No es aceptable que otro médico modifique o interrumpa el tratamiento antimicrobiano de un paciente

◯ Muy de acuerdo
◯ De acuerdo
◯ En desacuerdo
◯ Muy en desacuerdo

30. No es recomendable que un farmacéutico sugiera un cambio del antimicrobiano

◯ Muy de acuerdo
◯ De acuerdo
◯ En desacuerdo
◯ Muy en desacuerdo

31. En mi institución, la retirada de antimicrobianos no es aceptada hasta completar un número predeterminado de días

◯ Muy de acuerdo
◯ De acuerdo
◯ En desacuerdo
◯ Muy en desacuerdo

32. La decisión de interrumpir un antimicrobiano casi siempre debe tener el visto bueno de mi jefe

◯ Muy de acuerdo
◯ De acuerdo
◯ En desacuerdo
◯ Muy en desacuerdo

33. La selección del antimicrobiano a prescribir es una decisión compartida

◯ Muy de acuerdo
◯ De acuerdo
◯ En desacuerdo
◯ Muy en desacuerdo

Por favor seleccione la respuesta correcta (puede marcar más de una opción)

34. En el protocolo de tratamiento para el COVID-19 se incluyen antibióticos, ¿Por qué?

- ◯ a. Para eliminar el coronavirus.
- ◯ b. Para tratar infecciones secundarias
- ◯ c. Para prevenir infecciones colaterales
- ◯ d. Ninguna es correcta

IV ACEPTACIÓN A EQUIPO INTERDISCIPLINARIO

35. ¿Considera que los médicos están dispuestos a recibir retroalimentación de otros profesionales en salud para optimizar la prescripción de los antimicrobianos?

- ◯ Muy de acuerdo
- ◯ De acuerdo
- ◯ En desacuerdo
- ◯ Muy en desacuerdo

36. Del siguiente listado de profesionales en salud, establezca el orden de quién estaría más dispuesto el médico a recibir retroalimentación al momento de prescribir un antimicrobiano (De muy dispuesto a nada dispuesto).

	Muy dispuesto	Algo dispuesto	Poco dispuesto	Nada dispuesto
Enfermera	◯	◯	◯	◯
Farmacéutico	◯	◯	◯	◯
Microbiólogo	◯	◯	◯	◯
Infectólogo	◯	◯	◯	◯

37. Si un equipo multidisciplinario de enfermedades infecciosas como parte de una estrategia, revisa la terapia antibiótica y le sugiere una modificación, ¿Considera que estaría dispuesto a considerar la modificación?

- ◯ Muy de acuerdo
- ◯ De acuerdo
- ◯ En desacuerdo
- ◯ Muy en desacuerdo

V RESISTENCIA ANTIMICROBIANA

38. Los antimicrobianos son sobre utilizados en los hospitales de Guatemala

- ◯ Muy de acuerdo
- ◯ De acuerdo
- ◯ En desacuerdo
- ◯ Muy en desacuerdo

39. Los antimicrobianos pueden dejar de funcionar en algún futuro

- ◯ Muy de acuerdo
- ◯ De acuerdo
- ◯ En desacuerdo
- ◯ Muy en desacuerdo

40. ¿Sabe qué es la resistencia antimicrobiana?

◯ Sí ◯ No

41. ¿En dónde y cuándo aprendió sobre la resistencia antimicrobiana?

42. Si contestó que sabe qué es la resistencia antimicrobiana, ¿Podría decirme en sus propias palabras qué es la resistencia antimicrobiana?

43. ¿Considera que desde que aprendió sobre la resistencia antimicrobiana sus hábitos de prescripción de antibióticos han cambiado?

◯ Si ◯ No

44. Considera que la resistencia antimicrobiana es un problema a nivel mundial?

◯ Sí ◯ No

45. ¿Considera que la resistencia antimicrobiana es un problema a nivel nacional?

◯ Sí ◯ No

46. ¿Considera que la resistencia antimicrobiana es un problema en su práctica diaria?

◯ Sí ◯ No

47. ¿Considera que su prescripción contribuye a generar resistencia antimicrobiana

◯ Sí ◯ No

Usted ha llegado al final del cuestionario, le agradecemos por su participación.
En la parte de abajo por favor indique dos opciones de día y hora en la que podamos contactarlo/a. Alguien del equipo de investigación se comunicará con usted para hacerle las preguntas abiertas del cuestionario. La llamada no tomará más de 20 minutos.

Dia 1

☐ Lunes ☐ Martes ☐ Miercoles ☐ Jueves ☐ Viernes

Hora en que prefiere ser contactado

☐ 10:00 a 12:00 ☐ 12:00 a 14:00 ☐ 14:00 a 16:00 ☐ 16:00 a 18:00

Dia 2

☐ Lunes ☐ Martes ☐ Miercoles ☐ Jueves ☐ Viernes

Hora en que prefiere ser contactado

☐ 10:00 a 12:00 ☐ 12:00 a 14:00 ☐ 14:00 a 16:00 ☐ 16:00 a 18:00
